# Continuous carbon dioxide flow via a modified 4-way stopcock to prevent hemostatic spray catheter clogging

**DOI:** 10.1016/j.vgie.2026.02.003

**Published:** 2026-02-19

**Authors:** Mina Alkomos, Dhruv Patel, Islam Rajab, Yana Cavanagh, Walid Baddoura

**Affiliations:** 1 Gastroenterology Department, St Joseph's University Medical Center, Paterson, New Jersey, USA; 2 Internal Medicine Department, St Joseph's University Medical Center, Paterson, New Jersey, USA

## Abstract

**Background and Aims:**

Clogging of the Hemospray catheter (TC-325 Hemospray; Cook Medical, Bloomington, Ind, USA) is a common technical challenge, particularly when the tip becomes wet during endoscopic procedures, leading to interruption of hemostasis. A prior solution described a modified airflow connection to prevent occlusion. We developed a simplified, reproducible method using commercially available carbon dioxide (CO_2_) insufflation devices and tubing, connected via a 4-way stopcock to provide continuous CO_2_ flow through the Hemospray catheter.

**Methods:**

The assembly incorporated commercially available CO_2_ insufflation devices and tubing, a 4-way stopcock, and the standard Hemospray system. Continuous CO_2_ flow was maintained throughout the procedure to displace moisture and preserve lumen patency.

**Results:**

Continuous CO_2_ flow effectively prevented catheter occlusion and maintained uninterrupted spray function even after fluid contact. The demonstration confirmed consistent delivery without clogging.

**Conclusions:**

This 4-way stopcock enables continuous CO_2_ flow through the Hemospray catheter, preventing clogging in a simple, low-cost, and reproducible manner. The set-up is readily adaptable to existing endoscopic systems and may enhance procedural efficiency and device reliability.

## Background

Clogging of the Hemospray catheter (TC-325 Hemospray; Cook Medical, Bloomington, Ind, USA) is a frequent technical problem, particularly when the catheter tip becomes wet during endoscopic procedures. Loss of spray function may interrupt hemostasis or require replacement of the delivery device. Prior work described a method to maintain airflow and prevent Hemospray catheter occlusion by directly connecting the tubing to the catheter, interchangeably with the Hemospray gun.^1^ In contrast, we propose a simplified, reproducible modification that uses commercially available carbon dioxide (CO_2_) insufflation devices and tubing, connected via a 4-way stopcock, to provide continuous CO_2_ flow through the Hemospray catheter. The 4-way stopcock allows the endoscopist to use any available tubing attached to their CO_2_ or air source, making this set-up adaptable to different units and equipment configurations.

**Figure 1:**
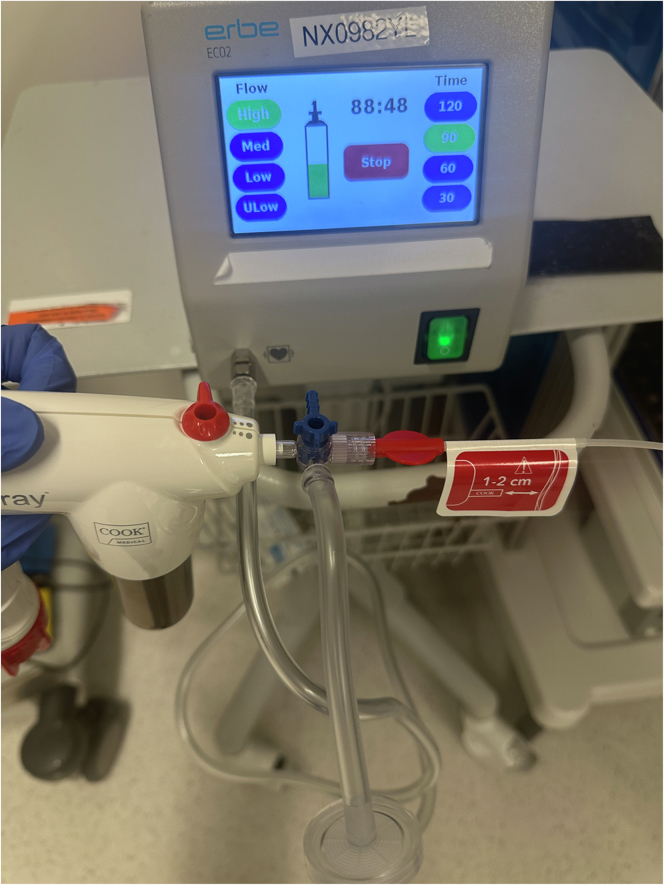
Final set-up. ECO_2_ insufflator (Erbe Elektromedizin GmbH, Tübingen, Germany).

**Figure 2:**
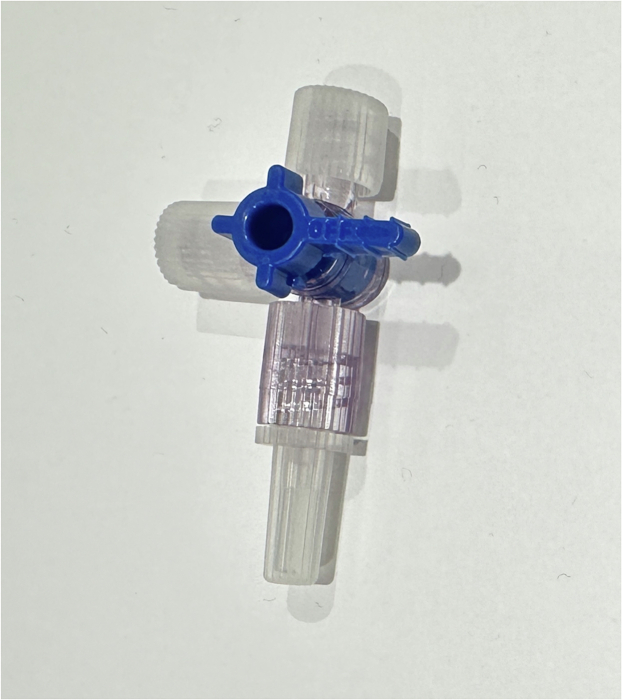
Discofix 4-way stopcock (B. Braun, Melsungen, Germany).

**Figure 3:**
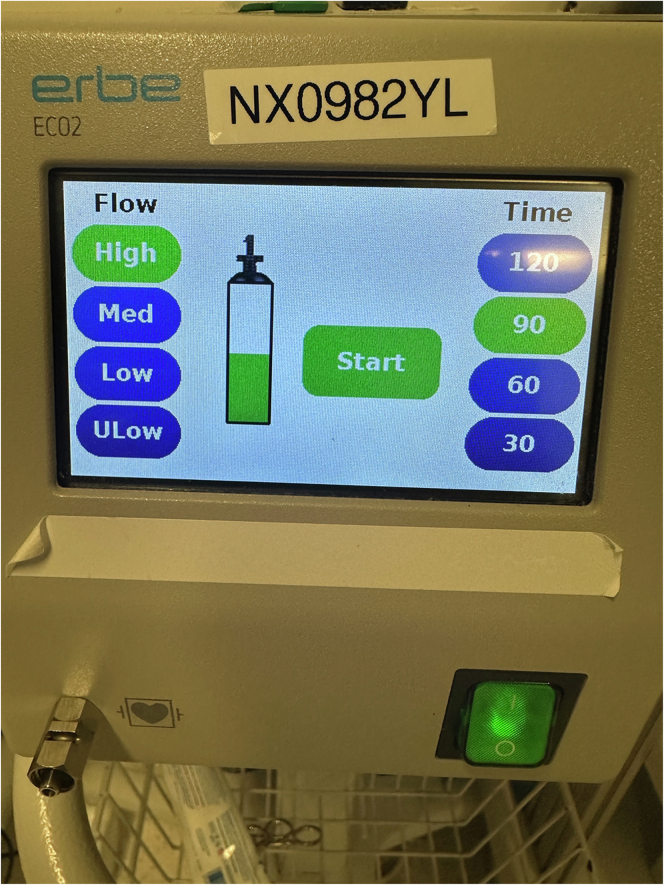
Erbe (Tübingen, Germany) ECO_2_ insufflator display with flow settings.

**Figure 4:**
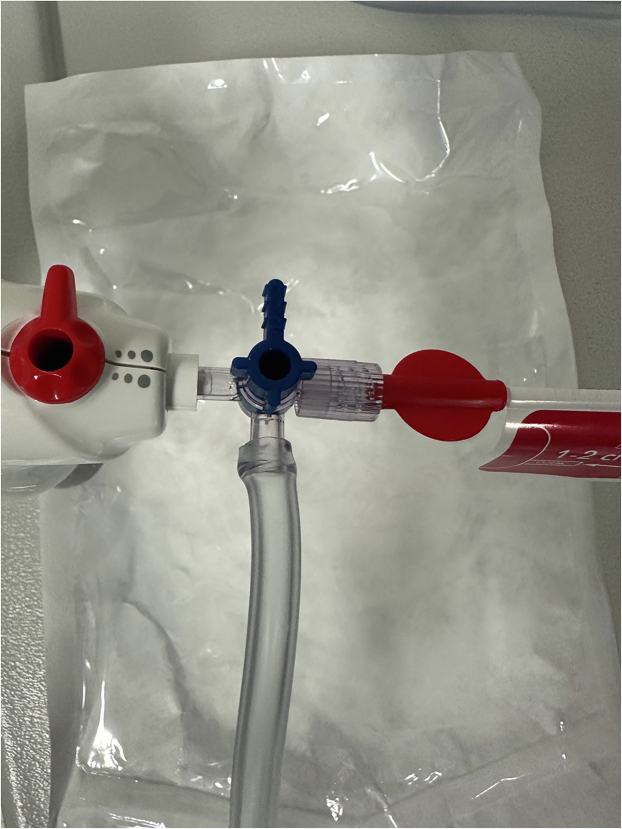
Four-way stopcock connected to Hemospray gun (Cook Medical, Bloomington, Ind, USA), carbon dioxide source, and the Hemospray catheter.

**Figure 5:**
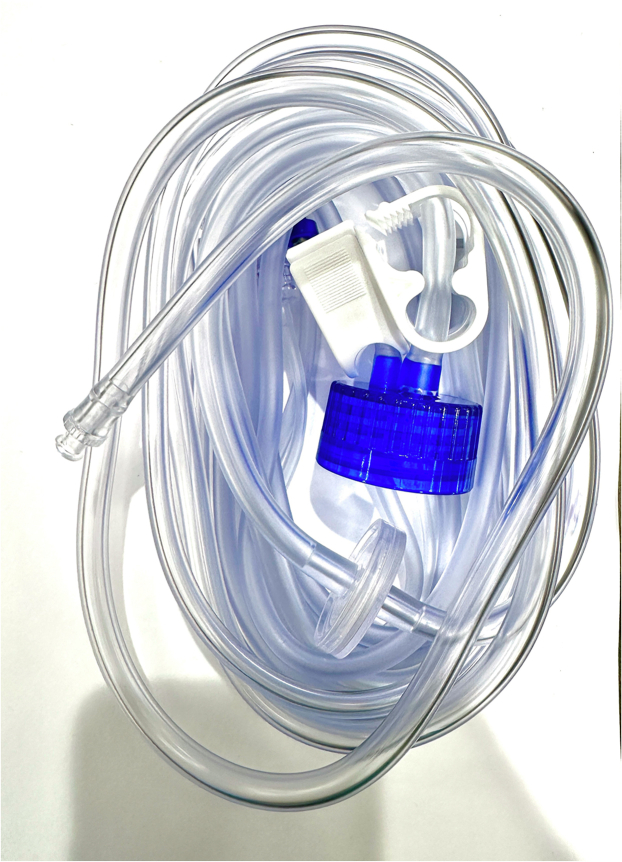
ERBEFLO (Erbe Elektromedizin GmbH, Tübingen, Germany) CleverCap tubing set.

The assembly consists of the following (Figure 1, Figure 2, Figure 3, Figure 4, Figure 5): (1) hybrid CO_2_ tubing set (Fig. 5), (2) Discofix (B. Braun, Melsungen, Germany) 4-way stopcock (Fig. 2), (3) Erbe (Erbe Elektromedizin GmbH, Tübingen, Germany) CO_2_ insufflator (ECO_2_) (Fig. 3), and (4) the standard Hemospray delivery system (Fig. 4).

### Steps

1. Connect the available CO_2_ tubing to the CO_2_ output port of the ECO_2_ unit on 1 side and the 4-way stopcock (Fig. 2) on the other side after modifying it to fit if needed.
2. Connect the Hemospray gun to the second port and the Hemospray catheter (Fig. 4) to the third port.
3. Set the ECO_2_ to high or medium flow and activate continuous CO_2_ output.

This configuration delivers a steady CO_2_ stream through the catheter, displacing moisture and preventing powder aggregation. During Hemospray activation, continuous flow maintained patency and allowed uninterrupted spraying (Fig. 1).

In this set-up, CO_2_ is routed directly to the Hemospray device rather than through the endoscope, preventing lens cleaning when CO_2_ flow is active. To permit lens irrigation, the operator should switch the device to air as needed or cycle the airflow on and off to minimize the risk of overinsufflation. In the event that Hemospray material enters the CO_2_ insufflation line, cycling the CO_2_ source off and on and allowing a brief flush interval will effectively clear the line.

## Results

With this set-up, Hemospray delivery remained continuous with no evidence of clogging, even after the catheter tip contacted irrigating fluid. The continuous CO_2_ system provided reliable flow control, and catheter patency was maintained throughout the application. A demonstration Video 1 (available online at www.videogie.org) confirms sustained spray function under continuous CO_2_ flow.

## Discussion

This modification provides a simple, low-cost solution using equipment available in most endoscopy units. The 4-way stopcock allows easy integration without altering the Hemospray system or interfering with endoscopic insufflation.

This approach may enhance procedural efficiency, reduce device waste, and improve control in challenging bleeding scenarios. By overcoming the inherent constraints of EndoClot's (EndoClot Plus, Inc, Santa Clara, Calif, USA) low-volume formulation and NexPowder (Next Biomedical Co, Ltd, Incheon, South Korea) clogging, the proposed modification capitalizes on Hemospray's higher-gram capacity and faster dispersion. This enhancement not only improves clinical performance but also provides a more favorable cost profile, placing the optimized Hemospray system at the forefront of available hemostatic technologies.

## Conclusion

A 4-way stopcock connected to any commercially available CO_2_ insufflation device and tubing effectively prevents Hemospray catheter clogging. The set-up is simple, reproducible, and adaptable to existing endoscopic systems.

## Disclosure

The following author disclosed financial relationships: Y. Cavanagh: Consultant for Boston, Olympus, and Swan Endosurgical Inc. All other authors disclosed no financial relationships.

## Supplementary data

Video **Video 1. Device assembly and in vitro application**. This video demonstrates the assembly and in vitro use of a 4-way stopcock–modified hemostatic powder delivery system. The 4-way stopcock is connected to the hemostatic powder catheter and a continuous carbon dioxide source, allowing controlled airflow management. The catheter is advanced through the endoscope working channel, and powder deployment is demonstrated within a water-filled sample container to simulate a fluid-rich environment. The video highlights continuous, uninterrupted powder delivery without catheter clogging, as well as the ability to temporarily divert airflow to allow endoscopic lens cleaning without disconnecting or replacing the catheter. **Video 2. In vivo application for active upper gastrointestinal bleeding**. This video demonstrates in vivo application of the 4-way stopcock–assisted hemostatic powder delivery system in a 62-year-old male presenting with active upper gastrointestinal bleeding. Esophagogastroduodenoscopy revealed a bleeding lesion suspicious for malignancy along the lesser curvature extending from the gastroesophageal junction, which was biopsied and associated with ongoing bleeding. Hemostatic powder was applied using the continuous carbon dioxide 4-way stopcock configuration, resulting in effective hemostasis without catheter clogging or interruption of powder delivery, and no need for catheter exchange or flushing during the procedure.
